# [177Lu]Lu-DOTATATE Molecular Radiotherapy During Induction Chemotherapy for First-Line Stage 4 High-Risk Neuroblastoma in South African Children: Protocol for a Phase 1 Randomized Controlled Trial (LuDO-SA Trial)

**DOI:** 10.2196/93547

**Published:** 2026-09-30

**Authors:** Jaques van Heerden, Mariana Kruger, Kershinee Reddy, Anel van Zyl, James Warwick, Celeste Burger, Tumelo Moalosi, Christoffel van Reenen, Alan Davidson, Khanyisile Hlongwa, Daniella Violante, Nanette Bruwer, Gita Naidu, Biance Rowe, Lizette Louw, Bronwin van Wyk, Mariza Vorster, Thokozani Mkhize, Anita Brink

**Affiliations:** 1 Department of Paediatrics and Child Health, Faculty of Medicine and Health Sciences, Tygerberg Hospital, Stellenbosch University, Stellenbosch University, Tygerberg Campus, Francie van Zijl Drive, Cape Town, Western Cape, 7505, South Africa, 27 219389506; 2 Department of Paediatric Haemato-Oncology, MIPRO laboratory, Antwerp University Hospital, University of Antwerp, Antwerp Province, Belgium; 3 School of Psychology, University of KwaZulu-Natal, Pietermaritzburg, KwaZulu-Natal, South Africa; 4 Faculty of Medicine and Health Sciences, University of Antwerp, Antwerp Province, Belgium; 5 Department of Paediatric Haemato-Oncology, Department of Paediatrics and Child Health, Inkosi Albert Luthuli Central Hospital, University of Kwa-Zulu Natal, Durban, KwaZulu-Natal, South Africa; 6 Department of Nuclear Medicine, Faculty of Medicine and Health Sciences, Tygerberg Hospital, Stellenbosch University, Cape Town, Western Cape, South Africa; 7 Division of Paediatric Haematology and Oncology, Red Cross War Memorial Children’s Hospital, Department of Paediatrics and Child Health, University of Cape Town, Cape Town, Western Cape, South Africa; 8 Division of Nuclear Medicine, Red Cross War Memorial Children’s Hospital, Department of Paediatrics and Child Health, University of Cape Town, Cape Town, Western Cape, South Africa; 9 Department of Nuclear Medicine, Groote Schuur Hospital, University of Cape Town, Cape Town, Western Cape, South Africa; 10 Department of Paediatric Haemato-Oncology, Department of Paediatrics and Child Health, University of the Witwatersrand, Chris Hani Baragwanath Academic Hospital, Johannesburg, Gauteng, South Africa; 11 Center of Molecular Imaging and Theranostics, Johannesburg, Gauteng, South Africa; 12 Department of Medical Physics, Dr George Mukhari Academic Hospital, Sefako Makgatho University, Ga-Rankuwa, Gauteng, South Africa; 13 Department of Nuclear Medicine, Inkosi Albert Luthuli Central Hospital, University of KwaZulu Natal, Durban, Kwa-Zulu Natal, South Africa; 14 Nuclear Medicine and Diagnostic Imaging Section, Division of Human of Health, Department of Nuclear Sciences and Applications, International Atomic Energy Agency, Vienna, Austria

**Keywords:** [^177^Lu]Lutetium DOTATATE, peptide receptor radionuclide therapy, PRRT, high-risk, neuroblastoma, first-line therapy, radiopharmaceutical, metastatic remission, South Africa

## Abstract

**Background:**

In South Africa, the metastatic response rate for stage 4 high-risk neuroblastoma (HR-NB) to induction chemotherapy is lower than that of similar regimens in well-resourced settings. Augmenting current induction chemotherapy regimens with molecular radiotherapy, without increasing toxicity, is an attractive systemic treatment in radioisotope-avid tumors, especially in a resource-limited setting where stem cell transplantation and immunotherapy are unavailable and costly. Clinical trials of [^177^Lu]Lutetium DOTATATE ([^177^Lu]Lu-DOTATATE) have primarily focused on refractory or relapsed HR-NB, with disappointing results. This study evaluates the feasibility and dose-limiting toxicity grading of adding a single dose of [^177^Lu]Lu-DOTATATE to induction chemotherapy in achieving metastatic remission rates.

**Objective:**

The aim of the proof-of-concept, open-label study is to evaluate the safety and efficacy of induction [^177^Lu]Lu-DOTATATE peptide receptor radionuclide therapy (PRRT) on postinduction response rate in children diagnosed with HR-NB. The primary objectives are to evaluate the dose-limiting toxicity profile of participants treated with induction [^177^Lu]Lu-DOTATATE PRRT in conjunction with standard induction chemotherapy; to evaluate the dosimetry in South African patients receiving induction [^177^Lu]Lu-DOTATATE PRRT; and to evaluate the feasibility related to resources in patients treated with induction [^177^Lu]Lu-DOTATATE PRRT in conjunction with standard induction chemotherapy. The secondary objective is to determine the postinduction metastatic response rate in patients diagnosed with HR-NB after receiving induction [^177^Lu]Lu-DOTATATE PRRT in conjunction with standard induction chemotherapy.

**Methods:**

The LuDO-SA trial is a phase 1, open-label, multicenter, 2-arm, randomized controlled clinical trial. Children aged 18 months to 18 years are eligible. The experimental arm, consisting of a single dose of [^177^Lu]Lu-DOTATATE after cycle 1 of OJEC (vincristine, carboplatin, etoposide, and cyclophosphamide) and OPEC (vincristine, cisplatin, etoposide, and cyclophosphamide) chemotherapy, will be compared to the standard arm of OJEC and OPEC chemotherapy. The postinduction metastatic response rate is based on the International Neuroblastoma Response Criteria (INRC). The statistical analysis will be descriptive. The Fisher exact test will be used to evaluate the significance of end-of-trial outcomes.

**Results:**

The trial received institutional ethics approval from 4 South African universities in 2022 and SAHPRA (South African Health Products Regulatory Authority) regulatory approval. The trial, funded by Kinderkankerfonds vzw and the UZA Foundation, commenced recruitment in January 2023. As of July 2026, a total of 15 patients have been enrolled across 4 academic hospital sites. Recruitment is projected to be completed by June 2027, with results expected to be published in December 2027.

**Conclusions:**

In this paper, we present the protocol of the LuDO-SA trial. The rationale and design of the trial in a resource-limited setting and its use in first-line regimens are discussed, and the current status of international [^177^Lu]Lu-DOTATATE molecular radiotherapy trials is summarized.

## Introduction

### Global Treatment and Survival Disparities

The management of childhood malignancies has attained overall survival (OS) rates of up to 80% in many tumors [1]. This is partly due to the progress in treatment possibilities. In low- and middle-income countries (LMICs), the outcomes are less favorable mainly because treatment options are not as diverse as in high-income countries (HICs) [1]. Neuroblastoma is a tumor that, despite these advances, has only attained an OS rate of up to 60% in HICs [2]. Survival in LMICs is poor, with fewer treatment possibilities than in HICs [2]. In South Africa, the 2-year OS rate for high-risk neuroblastoma (HR-NB) is 27.1%, with long-term outcomes being even lower [3]. To improve disease outcomes, new and innovative applications of available treatments should be evaluated while being aware of resource limitations.

### Epidemiological Context

Neuroblastoma, a neuroendocrine tumor of the sympathetic nervous system, accounts for 7% to 8% of pediatric cancer deaths worldwide [4]. High-risk disease (HR-NB), driven by metastatic spread and adverse biology, represents roughly 50% of cases in HICs but up to 80% in LMICs [3,4]. This imbalance drives a stark survival gap: 5-year OS rates reach 60% to 70% in HICs with full multimodal therapy (chemotherapy, surgery, radiotherapy, autologous stem cell transplant, and anti-GD2 immunotherapy) but remains as low as 10% in LMICs, where these modalities are frequently unavailable [1]. In South Africa, specifically, retrospective data show that the majority of children present with HR-NB, and a substantial proportion require palliative rather than curative-intent management [3,5].

### Limitations of Current Induction Chemotherapy

Two sequential goals define HR-NB management: achieving sufficient metastatic remission during induction chemotherapy to permit local therapies (surgery or radiotherapy) and then consolidating that remission [1,2]. In HICs, standard induction regimens (rCOJEC [rapid cisplatin, vincristine, etoposide, cyclophosphamide, and carboplatin] or doxorubicin-containing protocols) achieve metastatic complete remission (mCR) in 36% to 37% of patients [6]. In the South African cohort, mCR was achieved in only 30.7% of patients with HR-NB [3]. In HICs, mCR is not necessary to proceed to local treatment because subsequent treatment steps, such as high-dose chemotherapy and maintenance treatment with immunotherapy, may eradicate remaining tumor cells [2,7]. Yet, high-dose chemotherapy and immunotherapy are not available to the majority of South African patients [3]. Clinicians remain reluctant to proceed to surgery without at least a very good partial response, limiting progression to definitive local therapy [7]. Escalating chemotherapy intensity (eg, extended topotecan-vincristine-doxorubicin induction [8]) adds modest gains in mCR (5%‐10%) at the cost of increased toxicity, without a clear survival benefit [2]. Anti-GD2 immunotherapy improves survival by 15% to 20% in HICs [2] but is prohibitively expensive (approximately €210,000 [US $245,000] per treatment course) [7,9] and is not accessible in South African public-sector hospitals. Autologous stem cell transplant is similarly constrained by resource availability. There is, therefore, a clear, unmet need for an induction strategy that can improve mCR rates without depending on infrastructure or agents that are inaccessible in this setting.

### Rationale for [^177^Lu]Lu-DOTATATE as an Induction Adjunct

Neuroblastoma is highly radiosensitive [10], and up to 95% to 98% of tumors express somatostatin receptors (SSTRs) amenable to [^68^Ga]^68^Gallium DOTA-peptide ([^68^Ga]Ga-DOTA-peptide) imaging and [^177^Lu]Lutetium DOTATATE ([^177^Lu]Lu-DOTATATE) peptide receptor radionuclide therapy (PRRT) [11,12]. [^131^I]Iodine metaiodobenzylguanidine ([^131^I]I-mIBG) therapy, the existing radionuclide option in South Africa, is already used as an induction adjunct but carries significant hematological toxicity, requires prolonged radiation-shielded inpatient admission, excludes approximately 10% of mIBG-nonavid tumors, and depends on an inconsistent local isotope supply [2,5,13-15]. [^177^Lu]Lu-DOTATATE PRRT, by contrast, has a more favorable early safety profile (reversible grade 2‐3 hematological toxicity, minimal renal toxicity, outpatient administration), targets a receptor expressed in a larger proportion of tumors, and is comparatively less resource-intensive to deliver [11,12,16-18]. A full comparison of radiopharmacology, administration protocols, safety profiles, and cost is provided in Table S1 of Multimedia Appendix 1. Given these features, [^177^Lu]Lu-DOTATATE PRRT, used as an adjunct to standard induction chemotherapy rather than as monotherapy, represents a plausible, more accessible strategy to improve mCR rates in South African children with HR-NB.

### The Evidence Gap for First-Line Use of [^177^Lu]Lu-DOTATATE

Existing clinical experience with [^177^Lu]Lu-DOTATATE PRRT in neuroblastoma is confined to the relapsed or refractory setting, in small cohorts of heavily pretreated patients, and shows encouraging but inconsistent responses [12,16,17]. No published trial has evaluated [^177^Lu]Lu-DOTATATE PRRT in previously untreated, newly diagnosed children. A UK phase 2a study of empiric-dose, 4-cycle monotherapy in relapsed or refractory disease reported no objective responses, and dosimetric follow-up work showed that tumor-absorbed dose falls sharply across repeated cycles while renal dose remains stable, which implies that low-intensity, repeated-cycle monotherapy progressively underdoses the tumor [19,20]. Where clinical benefit has been observed, it has come from combination approaches: Fathpour et al [21] reported partial or complete responses in 3 of 5 children treated with [^177^Lu]Lu-DOTATATE PRRT alongside intensive chemotherapy in a relapse or refractory setting. A Chinese cohort using higher dose per-cycle activity (100‐200 MBq/kg) reported disease control in approximately 55% of children with no renal toxicity [22]. Ongoing trials (eg, the LuDO-N study) are testing dose-intensified, dosimetry-guided monotherapy schedules, but outcome data are not yet available [23]. Taken together, the literature supports 2 consistent points: single-cycle or combination use, rather than repeated low-dose monotherapy, is likely to be more effective, and no data yet exist on [^177^Lu]Lu-DOTATATE PRRT administered as part of first-line induction. This represents the specific gap this study addresses. By limiting [^177^Lu]Lu-DOTATATE PRRT to a single administration, the trial aims to prevent treatment delays but also to evaluate the toxicity in combination with OJEC (vincristine, carboplatin, etoposide, and cyclophosphamide) and OPEC (vincristine, cisplatin, etoposide, and cyclophosphamide) in previously untreated children.

### Aims and Objectives

We hypothesize that adding a single cycle of induction [^177^Lu]Lu-DOTATATE PRRT to standard first-line chemotherapy is feasible and safe and may improve mCR rates in South African children with newly diagnosed stage 4 HR-NB, thereby increasing the proportion of children able to proceed to definitive local therapy. This proof-of-concept pilot study aims to evaluate the feasibility, safety, and preliminary efficacy, with mCR as the end point of a single-cycle induction [^177^Lu]Lu-DOTATATE PRRT combined with standard chemotherapy in this setting.

### Objectives

The primary objectives are to evaluate the dose-limiting toxicity (DLT) profile of participants treated with induction [^177^Lu]Lu-DOTATATE PRRT in conjunction with standard induction chemotherapy; to evaluate dosimetry in South African patients receiving induction [^177^Lu]Lu-DOTATATE PRRT; and to evaluate the resource-related feasibility in patients treated with induction [^177^Lu]Lu-DOTATATE PRRT in conjunction with standard induction chemotherapy.

The secondary objective (exploratory objective) is to determine the postinduction metastatic response rate in patients diagnosed with HR-NB after receiving a single cycle of induction [^177^Lu]Lu-DOTATATE PRRT in conjunction with standard induction chemotherapy based on the International Neuroblastoma Response Criteria (INRC).

## Methods

### Study Design

Eligible participants are enrolled across 4 participating academic hospital sites in South Africa (Tygerberg Hospital, Red Cross War Memorial Children’s Hospital, Chris Hani Baragwanath Academic Hospital, and Inkosi Albert Luthuli Central Hospital). Following confirmation of eligibility, participants are allocated in a 1:1 ratio to either the standard or experimental arm using a computer-generated permuted-block randomization sequence with randomly varying block sizes of 4 and 6. Randomization is performed centrally by the national principal investigators (PIs), with allocation concealed from site investigators and participants until after enrollment. Participants in the standard arm receive 7 cycles of OJEC and OPEC induction chemotherapy per the standard South African protocol. Participants in the experimental arm receive a single intravenous administration of [^177^Lu]Lu-DOTATATE PRRT (3.5‐5.5 GBq; approximately 0.4 GBq/kg), administered following completion of cycle 1 of OJEC and OPEC, with subsequent cycles 2 to 7 proceeding as per the standard arm. Response assessment is conducted at the end of induction (after cycle 7) in all participants using [^68^Ga]Ga-DOTATATE PET (positron emission tomography)/CT (computed tomography); CT of the thorax, abdomen, and pelvis; and bone marrow trephine biopsy, with responses based on the 2017 INRC (Multimedia Appendix 2). A schematic of the trial design is presented in Figure 1.

**Figure 1.**
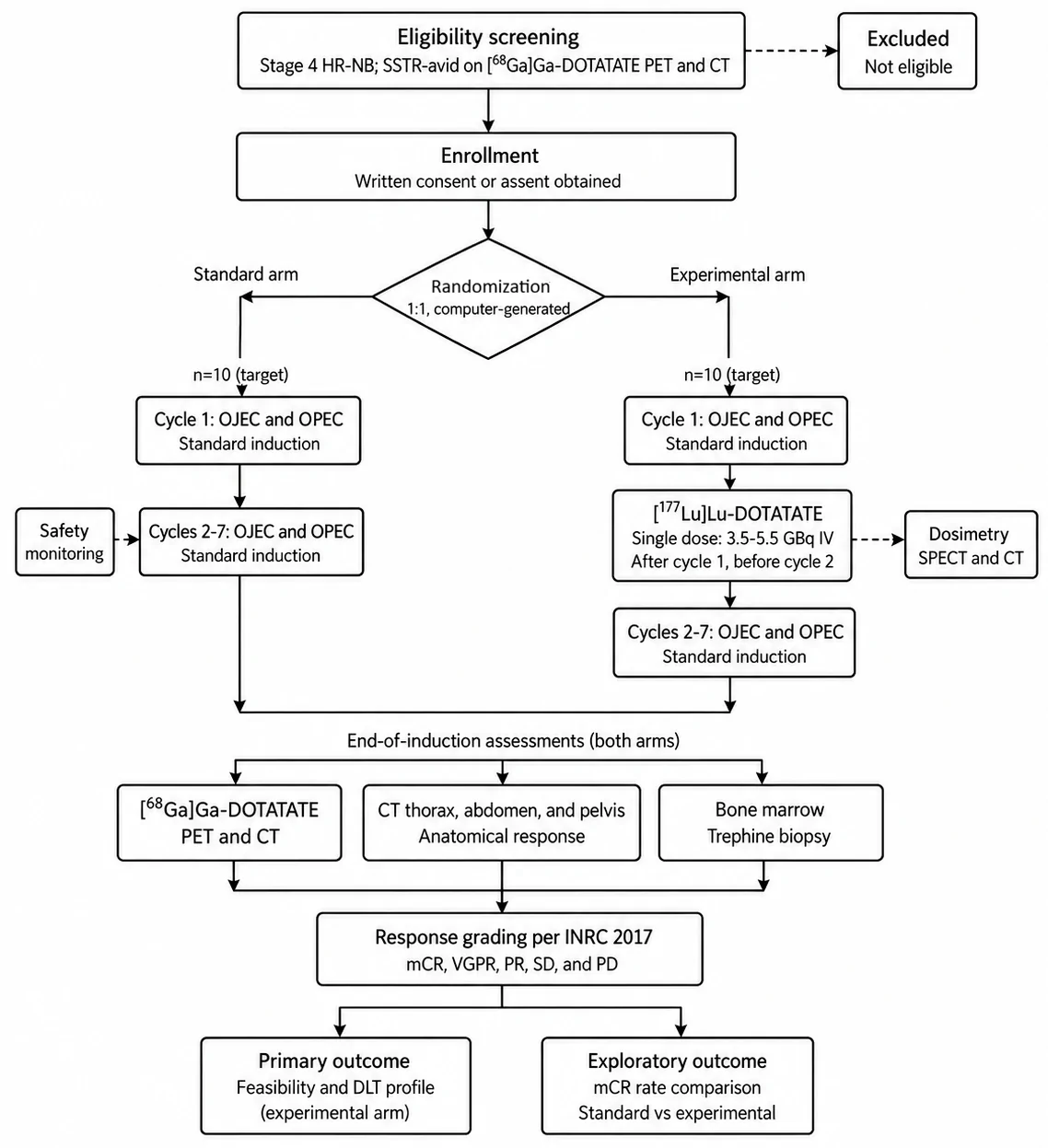
LuDO-SA trial schematic CONSORT (Consolidated Standards of Reporting Trials)-style diagram (numbers are not shown as recruitment is ongoing). CT: computed tomography; DLT: dose-limiting toxicity; HR-NB: high-risk neuroblastoma; INRC: International Neuroblastoma Response Criteria; mCR: metastatic complete remission; OJEC: vincristine, carboplatin, etoposide, and cyclophosphamide; OPEC: vincristine, cisplatin, etoposide, and cyclophosphamide; PD: progressive disease; PET: positron emission tomography; PR: partial remission; SD: stable disease; SPECT: single-photon emission computed tomography; SSTR: somatostatin receptor; VGPR: very good partial response.

### Definitions

The standard of care for neuroblastoma management is defined as management according to the national prospective management protocol SACCSG (South African Children’s Cancer Study Group) NB-2017.

Induction [^177^Lu]Lu-DOTATATE PRRT is defined as a single administration (including diagnostics and prophylaxis) of [^177^Lu]Lu-DOTATATE PRRT.

Medical exclusion is defined as the exclusion of any significant sites of active disease, as determined by unequivocal, contrast-enhancing lesions on CT or magnetic resonance imaging that lack SSTR expression, which can be confirmed by a 2-[^18^F]fluorodeoxyglucose PET/CT, if available.

DLT is defined as any grade 3, 4, or 5 hematological or nonhematological side effect according to the Food and Drug Administration’s Common Terminology Criteria for Adverse Events (CTCAE).

### Study Design

The study will be a phase 1, multicenter, prospective, open-label, 2-arm safety study. This protocol was developed and is reported in accordance with the SPIRIT (Standard Protocol Items: Recommendations for Interventional Trials) guidelines (Checklist 1).

### Study Setting

The pediatric hematology and oncology units and associated nuclear medicine departments that are participating in the study are Red Cross War Memorial Children’s Hospital, Cape Town; Tygerberg Academic Hospital, Cape Town; Chris Hani Baragwanath Academic Hospital, Johannesburg; and Inkosi Albert Luthuli Academic Hospital, Durban.

All 4 sites are accredited to deliver standard-of-care chemotherapy treatment and radioisotope therapy, as well as biochemical and radiological treatment monitoring services.

### Study Population and Sampling

Patients diagnosed with stage 4 HR-NB according to the SACCSG NB-2017 national protocol will be eligible to be recruited prospectively and centrally and electronically randomized to either the [^177^Lu]Lu-DOTATATE PRRT arm or the standard-of-care arm. Therefore, proactive recruitment is not possible. The inclusion and exclusion criteria are given in Textbox 1 [24-26].

The study will randomize 10 patients to each of the standard and experimental (interventional) arms. Thus, a total of 20 patients will be included in the study (see the SPIRIT figure in Multimedia Appendix 3).

Textbox 1. **Inclusion and exclusion criteria.**

**Inclusion criteria**

- All patients aged 18 months to 18 years with
  - Biopsy-proven high-risk neuroblastoma (HR-NB)
  - Radiologically reported neuroblastoma with positive u-catecholamine levels (homovanillic acid [HVA] and vanillylmandelic acid [VMA])
  - Bone marrow and trephine (BMAT) with involvement of neuroblastoma and positive u-catecholamine levels (HVA and VMA), in cases where a biopsy is not possible or radiological evidence is inconclusive
- Patients who have not previously been on treatment
- Neuroblastoma disease sites should demonstrate avid uptake of [^68^Ga]Ga-DOTA-peptide that is greater than uptake in the liver

**Exclusion criteria**

- Patients who have been previously treated with chemotherapy or radiolabeled somatostatin analogues
- Patients with indeterminate or inconclusive histology results or BMAT and u-catecholamine levels (HVA and VMA) to diagnose neuroblastoma
- Poor-quality investigations or investigations that provide incomplete information for study purposes
- Consent or assent for treatment has not been granted
- Medical exclusion criteria for [^177^Lu]Lu-DOTATATE peptide receptor radionuclide therapy (PRRT)
  - Absolute contraindication
    - Severe acute concomitant illnesses that prevent the safe administration of oncological treatment (see Multimedia Appendix 4 for definitions)
    - Severe, unmanageable psychiatric disorders
    - Renal function with a glomerular filtration rate (GFR) of <30 mL/min/1.73 m^2 ^or patients on dialysis for renal impairment
    - Patients who are pregnant
    - Patients with radiologically proven brain metastases (excluding the skull) or metastases protruding through the blood-brain barrier
  - Relative contraindication (exclusion based on a clinician’s assessment)
    - Renal function with a GFR of <50 mL/min/1.73 m^2^
    - Severely compromised bone marrow: neutrophils <1000/µL, absolute neutrophil count <1000/µL, platelets <75,000/µL, and hemoglobin <8 g/dL
    - Compromised liver function: total bilirubin level >3 times the upper limit of normal; serum albumin <3.0 g/dL
    - Patients with obstructive nephropathy diagnosed by a ^99m^Tc-mercaptoacetyltriglycine ([^99m^Tc]T-MAG3)-renogram; exclusion will be based on the degree of obstruction to prevent toxic radiation doses

### Timeline

The 4 hospitals account for nearly a third of the 30 to 40 patients diagnosed with neuroblastoma per year in South Africa. Therefore, we expect the study duration to be 3 to 4 years, starting when all institutional ethics committees and authorities have given approval to conduct the study.

### Study End Point

The end point of the study is the evaluation of the treatment toxicity profile. Anemia, thrombocytopenia, neutropenia, and infections during induction chemotherapy will serve as outcomes to evaluate the side-effect profile (see the data collection sheet “Toxicity” in Multimedia Appendix 5) [26].

An exploratory end point is the metastatic tumor response between the standard and [^177^Lu]Lu-DOTATATE PRRT arms after completion of the induction chemotherapy (after cycle 7 of chemotherapy). The measure of both study arms will be the INRC, and the radioisotope response criteria will be based on modified Curie scores.

### Clinical Methodology

#### Treatment Protocol

Patients will be managed according to the national SACCSG NB-2017 neuroblastoma treatment protocol. The standard arm induction chemotherapy is the internationally recognized OJEC and OPEC induction chemotherapy for HR-NB (Multimedia Appendix 6). The standard arm will also serve as the comparator.

All patients randomized to the experimental (interventional) arm will undergo diagnostic [^68^Ga]Ga-DOTA-peptide PET/CT imaging to confirm SSTR avidity to be eligible for [^177^Lu]Lu-DOTATATE PRRT. Simple randomization will be performed using a computer-generated randomization program. Patients in the experimental (interventional) arm will be admitted on a one-night inpatient admission for the [^177^Lu]Lu-DOTATATE PRRT. A full blood count, urea, creatinine, and liver function tests (minimum bilirubin and albumin) will be performed using peripheral blood to confirm that the starting requirements are met, independent of any blood tests performed 2 weeks prior (Multimedia Appendix 7).

In the event that diagnostic [^68^Ga]Ga-DOTA-peptide PET/CT imaging cannot be performed at diagnosis, the first cycle of chemotherapy may be given, with diagnostic [^68^Ga]Ga-DOTA-peptide PET/CT imaging performed no later than 2 weeks after chemotherapy.

#### Implementation

The local or institutional PIs will identify potential participants, perform screening, and announce the potential participant to the national PIs. The national PIs will verify the potential participant against inclusion criteria and, when eligible, proceed to randomize the participant. The national PIs will inform the local PI of the randomization. If the participant is randomized to the experimental (interventional) arm, the national PIs will also inform the producer of the [^177^Lu]Lu-DOTATATE isotopes to initiate logistics and delivery.

#### Allocation Concealment

None of the institutional PIs nor participants will have insight or access to the randomization process. Therefore, their enrollment and randomization of participants will be done by the national PIs in consecutive order. It will not be possible to blind the institutions or participants to the result of randomization due to the nature of the intervention itself.

#### Blinding of Treatment Response Evaluations

Response evaluation will be done by institutional specialists with expertise in scoring procedures for neuroblastoma based on the INRC. Each participant’s end-of-induction chemotherapy imaging will be scored by a second specialist, blinded to the primary scoring. The national PIs, also blinded to the scoring, will evaluate the scores and be the adjudicators if the scores are discrepant.

### Treatment

#### Pretreatment

Renal protective pretreatment with an arginine (25 g)/lysine (25 g) amino acid mixture at a rate of 7.5 mL/kg should be administered over 4 hours. The start of the infusion should precede the therapeutic dose of [^177^Lu]Lu-DOTATATE isotopes by at least 30 minutes [12,27].

#### Therapy

The recommended activity of [^177^Lu]Lu-DOTATATE will be a fixed dose of 3.5 to 5.5 GBq (approximately 0.4 GBq/kg) in 50 mL of normal saline administered over 15 minutes.

#### After Administration

Weekly full blood counts, urea and electrolyte tests, and liver function tests will be performed until induction chemotherapy commences in the third week after [^177^Lu]Lu-DOTATATE therapy.

Specific and nonspecific tumor markers will be determined to document disease response. The nonspecific tumor markers will include serum lactate dehydrogenase and ferritin. These will be determined weekly during follow-up. Specific tumor markers, homovanillic acid (HVA) and vanillylmandelic acid (VMA), will be determined in the urine at 6 to 8 weeks.

A posttherapy [^177^Lu]Lu-DOTATATE SPECT (single-photon emission computed tomography)/CT should be performed 24 hours after administration.

A second [^68^Ga]Ga-DOTATATE PET/CT scan will be performed 6 to 8 weeks after administration to assess disease response.

A third [^68^Ga]Ga-DOTATATE PET/CT scan will be performed at the end of induction chemotherapy to assess disease response, including metastatic remission status.

#### Adjuvant Chemotherapy Administration

After the administration of the [^177^Lu]Lu-DOTATATE therapy, weekly vincristine (1.5 mg/m^2^; maximum 2 mg per dose) will be administered until the start of induction chemotherapy (Multimedia Appendix 6).

### Treatment Protocol

#### Experimental (Interventional) Arm

To commence standard tumor treatment shortly after diagnosis, a single cycle of chemotherapy will be given according to the SACCSG NB-2017 protocol (SU HREC reference: S18/07/138). This therapy will initiate tumor control. During this period, all imaging and eligibility assessment for [^177^Lu]Lu-DOTATATE therapy will be done. After the first cycle, or in the event that the diagnostic [^68^Ga]Ga-DOTA-peptide PET/CT imaging has been delayed by 2 weeks, after cycle 2, the [^177^Lu]Lu-DOTATATE therapy will follow when the bone marrow has recovered, with absolute neutrophil count >1000 µL and platelets >75,000 µL. After [^177^Lu]Lu-DOTATATE therapy, weekly vincristine will be given until the bone marrow has recovered, which is expected from 4 to 6 weeks after administration of [^177^Lu]Lu-DOTATATE therapy, with absolute neutrophil count >1000 µL and platelets >100,000 µL. Thereafter, the induction chemotherapy can be continued (Figure 2).

**Figure 2.**
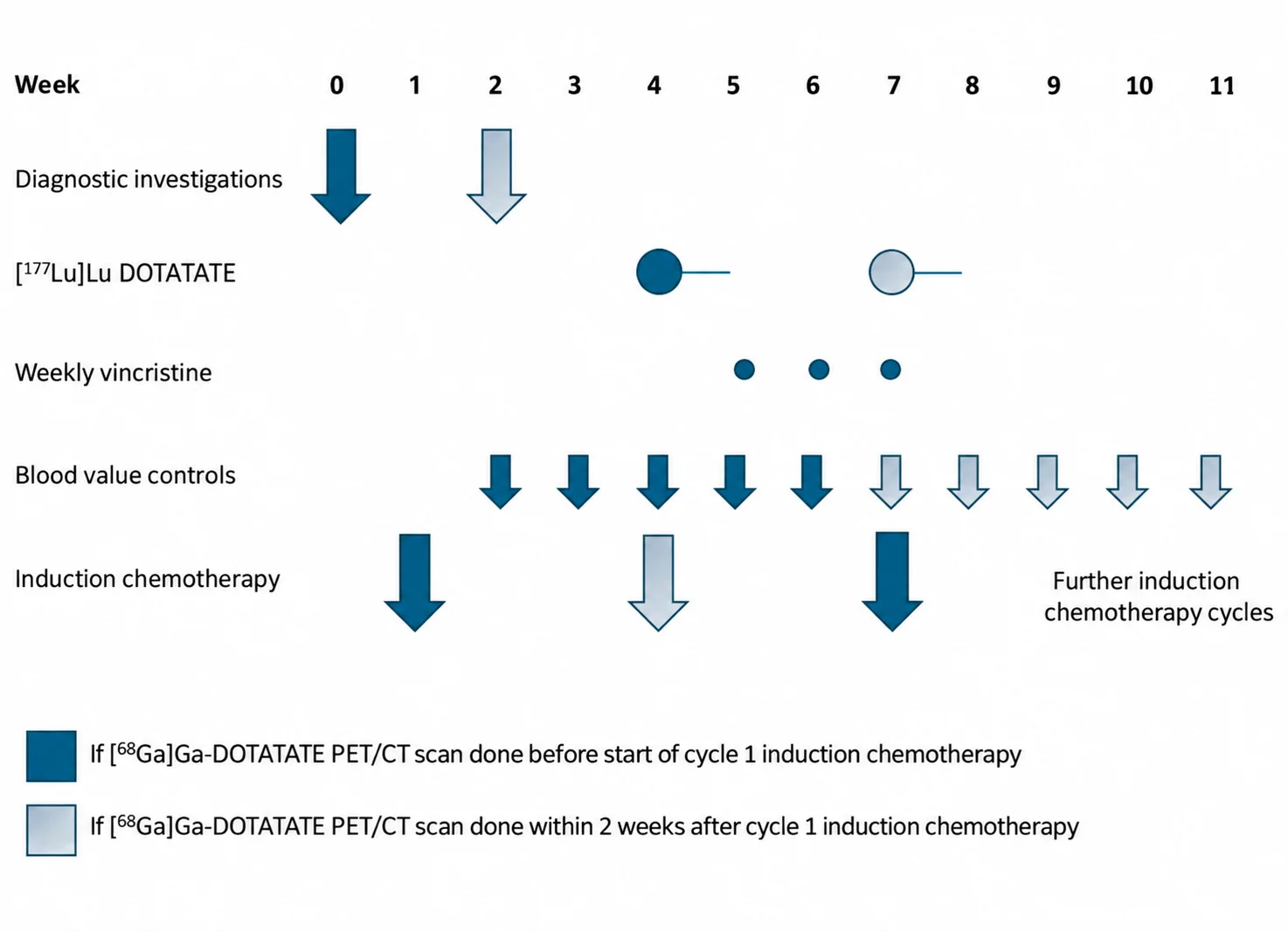
The treatment schedule for the experimental (interventional) arm. PET/CT: positron emission tomography/computed tomography.

#### Standard Arm

Patients in the standard arm will receive only chemotherapy according to the SACCSG NB-2017 protocol (Multimedia Appendix 6).

##### [^177^Lu]Lu-DOTATATE Dosimetry

Dosimetry data will be acquired for each therapy cycle [19,27,28]. SPECT/CT imaging should be performed for 4 postinjection time points, and the time points should be as close as possible to the following: 1 to 3 hours, 20 to 24 hours, 68 to 76 hours, and 144 to 196 hours. Internal dosimetry will be calculated for the kidneys and the spleen on HERMIA Voxel dosimetry (Hermes Medical Solutions; see full imaging procedure in Multimedia Appendix 8).

##### Metastatic Tumor Response Rate

Response assessment will be based on the SACCSG NB-2017 neuroblastoma management protocol and should include anatomic imaging for primary and metastatic soft-tissue disease, nuclear medicine imaging for assessment of soft-tissue and bone disease, and bilateral bone marrow aspirates and trephine biopsies for assessment of marrow and skeletal disease. The criteria are based on the INRC of 1993, the revised INRC criteria of 2017, and the soft-tissue Response Evaluation Criteria in Solid Tumors (RECIST) to accommodate the heterogeneous availability of diagnostic and imaging techniques (Multimedia Appendix 2).

### Criteria for Discontinuing

The experimental (interventional) arm is a single administration. Therefore, discontinuation will primarily be due to noninclusion based on exclusion criteria, if a participant withdraws consent before the intervention, and unacceptable DLT.

DLT is defined as any grade 3, 4, or 5 hematological or nonhematological adverse event according to the Food and Drug Administration’s CTCAE, judged by the investigator to be at least possibly related to study treatment. Safety monitoring will focus on the experimental (interventional) arm (OJEC and OPEC plus [^177^Lu]Lu-DOTATATE) therapy. Recruitment to the experimental (interventional) arm will be suspended and reviewed by the safety committee if more than 2 DLTs occur among the first 5 patients treated in this arm, or if 3 or more DLTs occur at any time among the first 10 patients treated in this arm. In this case, the safety committee will determine whether the regimen is unacceptably toxic. If so, accrual to the [^177^Lu]Lu-DOTATATE therapy arm will be permanently discontinued and the combination considered not suitable for further investigation at this dose and regimen.

### Adverse Event Reporting

All adverse events occurring from the administration of the study intervention until the completion of induction chemotherapy will be recorded and graded according to the CTCAE. Severity, expectedness, and relationship to the study treatment will be assessed by the treating local investigator and reported to the national PIs. Serious adverse events and suspected unexpected serious adverse reactions will be reported to SAHPRA (South African Health Products Regulatory Authority) and the institutional ethics committee according to South African Good Clinical Practice requirements. Participants experiencing an adverse event will be followed until resolution, stabilization, or determination of a clinically acceptable outcome.

### Concomitant Care

Concomitant care is the same in both study arms (Multimedia Appendices 2, 5 and 7). If there is an adverse reaction to the [^177^Lu]Lu-DOTATATE therapy, standard international care guidelines will be followed. If participants discontinue the allocated intervention but do not withdraw consent for data collection, protocol-specified safety and outcome assessments will continue wherever clinically feasible. The study has secured insurance to cover all costs and reimbursements in case of adverse events.

### Statistical Considerations

#### Study Sample

Taking into consideration that South Africa is a resource-limited setting, that stage 4 HR-NB is a rare disease in South Africa, and that this is an early-phase exploratory trial, the sample size is primarily based on feasibility and the need to obtain preliminary safety and response estimates rather than formal hypothesis testing. The aim is to enroll up to 20 participants in total, randomized 1:1 to a standard OJEC and OPEC induction chemotherapy-only arm and an OJEC and OPEC plus a single administration of [^177^Lu]Lu-DOTATATE experimental (interventional) arm. With 10 patients per arm, the study will provide descriptive estimates of the proportion of patients experiencing DLT and of early response based on modified Curie scores and INRG criteria (Multimedia Appendix 2) [29]. The limitation is that the resulting CIs will be wide. Therefore, if no DLTs are observed in 10 participants in an arm, the upper limit of the approximate 95% CI for the true DLT rate would be approximately 30%. This is sufficient to identify clearly unacceptable toxicities and provides justification for further evaluation in a larger study. The trial is therefore not powered to detect statistically significant differences in efficacy between the 2 arms but is designed to assess feasibility in this setting, characterize the safety profile of adding [^177^Lu]Lu-DOTATATE to the OJEC and OPEC induction chemotherapy, and explore potential benefits for larger trial populations.

#### Data Collection

Data will be collected by each individual participating unit and collated in a central, anonymous, electronic database in REDCap, managed by the PI, according to the data collection sheet (Multimedia Appendix 5). The data will be centralized in the Stellenbosch University OneDrive (Microsoft) facility database. Anonymity will be assured by awarding a unit-specific number to each patient. The list of patients with their unique number will be kept in a password-protected computer in a locked location. This information will only be known by the participating health care provider in each unit. The PI and statistician will only receive deidentified data with a site-specific identifier without knowledge of the patients’ identity. All of these measures will conform to the South African Protection of Personal Information (POPI) Act [30]. The national PIs will record screening results, failed screenings and reasons for the failed screening, nonadherence to protocols, and nonretention of patients. The national PIs will also ensure the completeness of data collection by regular follow-up of data collection and quality controls.

#### Data Analysis

The data will be analyzed using Stata (version 15.0; StataCorp LP) and the R statistical package (version 3.5; R Foundation for Statistical Computing). Categorical variables will be summarized as frequencies and percentages and continuous variables as the mean (SD) or the median (IQR), as appropriate. DLT and metastatic complete response rates will be reported with 95% CIs. Between-group differences in categorical outcomes, including mCR, will be explored using the Fisher exact test because of the small expected cell counts. Effect estimates with CIs will be emphasized rather than statistical significance. Participants who do not complete induction chemotherapy or the end-of-induction response assessment for reasons unrelated to treatment failure will be classified as nonevaluable and reported separately, with reasons documented. No imputation for missing response data is planned given the small sample size; per-protocol and intention-to-treat–analogous (all randomized) results will both be reported if they differ. Subgroup or adjusted analyses will only be done if they are of clinical interest as descriptive results only.

#### Timing of Data Analysis

Since this study is a pilot protocol for feasibility, analysis will only be done once a minimum of 7 participants in each study arm has been achieved. A safety and feasibility review alone will not trigger any efficacy-based stopping or protocol modification unless there are unacceptable safety concerns. With oversight approval, analysis will be done at the end of full study inclusion.

### Ethical Considerations

The study will conform to the ethical considerations described in the Declaration of Helsinki and the South African Good Clinical Practice: Clinical Trial Guidelines [31,32]. The study will also be done according to the standards for research in LMICs [31]. Both of these guidelines will ensure respectful research conduct toward study participants by the research team during the study period.

#### Ethics Committee Approval

Ethics approval was granted by the Stellenbosch University Human Research Ethics Committee (reference: M21/12/027); the University of Cape Town Human Research Ethics Committee (reference: 467/2022); the University of the Witwatersrand Human Research Ethics Committee (reference: R14/49); and the University of KwaZulu-Natal Biomedical Research Ethics Committee. Regulatory approval was granted by the SAHPRA (reference: 20220313).

#### Informed Consent

Written informed consent from caregivers, written informed assent from children aged 7 years or older, and willingness acknowledged for children younger than 7 years will be requested before study inclusion, with translation into the consenter’s primary language. Consent will be requested with the consenter’s right to withdraw without affecting care. No biological specimens will be collected, as this is not part of the informed consent.

#### Privacy and Confidentiality

Each participant receives a unique study number. All data are deidentified for analysis. Data are stored on password-protected systems compliant with the South African POPI Act (number: 4 of 2013).

#### Participant Compensation

Participants are compensated for trial inclusion and for each weekly visit during the trial period. The experimental arm will receive additional compensation for admission to administer the trial intervention. Compensation will be in accordance with the South African Department of Health and SAHPRA guidelines.

#### Cultural Sensitivity

All interventions and management recommendations will be done with consideration of the social and cultural aspects of each patient.

### Trial Monitoring

#### Data and Safety Monitoring Committee

An independent Data and Safety Monitoring Committee (DSMC) is responsible for reviewing accumulating safety data. The DSMC will be composed of a pediatric oncologist, a nuclear medicine physician, and a biostatistician, none of whom are site investigators or otherwise involved in the day-to-day conduct of the trial. They will convene an ad hoc review whenever a stopping-rule trigger is met, specifically, if more than 2 DLTs occur among the first 5 participants treated in the experimental (interventional) arm, or if 3 or more DLTs occur among the first 10 participants in that arm (*Criteria for Discontinuing* section). On review, the DSMC will determine whether the [^177^Lu]Lu-DOTATATE regimen is unacceptably toxic and has the authority to recommend suspension or permanent discontinuation of recruitment to the experimental arm. The DSMC operates independently of the sponsor and funders who have no role in this determination (*Trial Monitoring* section).

#### Radiation Safety Oversight

Two independent radiation experts, Prof James Larkin (Radiation and Health Physics Unit, University of the Witwatersrand) and Dr Stuart More (Department of Radiation Medicine, University of Cape Town), are appointed specifically to review cumulative radioisotope exposure and monitor radiation safety throughout the trial period, independent of the treating clinical teams. A review of dosimetry data will be done after the seventh participant has been treated in the experimental arm. Any safety concerns will be escalated to the DSMC or the ethics committees.

#### Independent Trial Monitoring

An independent monitor, unaffiliated with any of the 4 participating sites, will conduct remote monitoring of trial conduct and data quality once a minimum of 7 participants per arm have been enrolled and again at the end of the study period. The monitor reviews protocol adherence, data completeness, and consent documentation and reports findings directly to the national PIs and to participating sites’ ethics committees. Ad hoc monitoring will additionally be triggered by any reported protocol deviation or serious adverse event (*Trial Communications* section).

#### Auditing

The trial remains open to independent audit by any of the participating institutions’ ethics committees, SAHPRA, or funders at any time, on a yearly basis. This will be done by the site PIs.

## Results

### Trial Status

The LuDO-SA trial received ethics approval from Stellenbosch University (M21/12/027), the University of Cape Town (467/2022), the University of the Witwatersrand (R14/49), and University of KwaZulu-Natal and SAHPRA regulatory approval (reference: 20220313) in 2022.

### Recruitment

Recruitment commenced in January 2023 across 3 participating academic hospital sites, with the fourth starting recruitment in January 2025. As of July 2026, a total of 15 of the 20 planned participants have been enrolled: 7 in the standard arm and 8 in the experimental arm.

### Deviations

In January 2024 (version 2.0), the inclusion criteria were amended: [^68^Ga]Ga-DOTATATE PET/CT alone became sufficient for eligibility (previously both [^68^Ga]Ga-DOTATATE PET/CT and [^123^I]I-mIBG were required). [^123^I]I-mIBG avidity was removed as a criterion due to the limited and consistent availability of [^123^I]I-mIBG isotopes in South Africa, thereby excluding potential inclusions.

From the inclusion of the first patient, the medical physicists coordinated across the four sites to adapt the timing of the dosimetry scan in order to be logistically manageable during extended weekends, as well as due to limitations of staff availability and patient transportation requirements. The first draft protocol recommended exact SPECT/CT acquisitions at 2, 24, 96, and 160 hours. The timings were amended to the following time points: 1 to 3 hours postinjection, 20 to 24 hours, 68 to 76 hours, and 144 to 196 hours (when scanning is not interrupted by a weekend or public holiday); or 1 to 3 hours postinjection, 20 to 24 hours, 90 to 100 hours, and 144 to 196 hours (when scanning is interrupted by a weekend or public holiday; see the full imaging procedure in Multimedia Appendix 8).

### Timeline

Recruitment is projected to close by June 2027. Data analysis is anticipated to commence in the third quarter of 2027, with results expected to be published in the fourth quarter of 2027.

## Discussion

### Strengths and Limitations

A significant number of children in LMIC settings have limited treatment options for neuroblastoma and present with advanced disease. The poor metastatic tumor response during induction in South African patients has been reported by the study team under the main PhD study (SU HREC reference: S18/07/138 [PhD]; “The outcomes and management of neuroblastoma in South Africa”). Findings during this research have created the need to evaluate therapeutic options to improve the response rate of the tumor during induction. Research into neuroblastoma in South Africa is being continued under the main study as a post doc project (SU HREC reference: S18/07/138). This will be the first prospective study evaluating the role of [^177^Lu]Lu-DOTATATE therapy added to standard first-line chemotherapy for stage 4 HR-NB in South Africa and sub-Saharan Africa to improve mCR.

We anticipate that the addition of [^177^Lu]Lu-DOTATATE to standard OJEC and OPEC induction chemotherapy will be feasible within the constraints of a resource-limited, multisite South African setting and that its DLT profile will not exceed that of standard induction chemotherapy alone. Based on the mechanistic rationale for combining a radio-sensitizing or receptor-targeted agent with chemotherapy in a highly somatostatin receptor–avid tumor, we further anticipate a numerically higher end-of-induction mCR rate in the experimental arm than in the standard-of-care arm, although the trial is not powered to demonstrate this difference statistically. Confirmation of an acceptable DLT profile alongside a signal of improved mCR would support progression to a larger, adequately powered trial.

The LuDO-SA trial differs from existing [^177^Lu]Lu-DOTATATE neuroblastoma trials in 3 respects that are worth situating explicitly. First, published experience with [^177^Lu]Lu-DOTATATE in neuroblastoma has so far been confined to the relapsed or refractory setting in small, heavily pretreated cohorts [12,16,17]. The UK phase 2a trial of empiric-dose, 4-cycle [^177^Lu]Lu-DOTATATE monotherapy in relapsed or refractory disease reported no objective responses, and companion dosimetric work showed that tumor-absorbed dose falls sharply across repeated cycles while renal dose remained stable, suggesting that repeated low-dose monotherapy progressively underdoses the tumor rather than reflecting a lack of radiosensitivity [19,20]. Where clinical benefit has been reported, it has come from combination approaches: Fathpour et al [21] observed partial or complete responses in 3 of 5 children treated with [^177^Lu]Lu-DOTATATE alongside intensive chemotherapy. The LuDO-SA trial builds directly on this observation by testing a single, higher-activity dose delivered during first-line induction chemotherapy, rather than as sequential monotherapy after relapse. Second, the ongoing LuDO-N trial is evaluating dose-intensified, dosimetry-guided [^177^Lu]Lu-DOTATATE monotherapy schedules but in the relapsed or refractory rather than first-line setting, and outcome data are not yet available [23]. The LuDO-SA trial is, to our knowledge, the only trial evaluating [^177^Lu]Lu-DOTATATE as part of first-line induction in previously untreated children. Third, and specific to the South African and LMIC context, the LuDO-SA trial responds to a documented gap in first-line outcomes: 2-year OS rates for HR-NB in South Africa is markedly lower than in high-income settings (27.1% vs up to 60%) [2,3], at a time when stem cell transplantation and immunotherapy, both used to intensify first-line treatment in HICs, remain largely unavailable or unaffordable locally. [^177^Lu]Lu-DOTATATE’s more favorable early safety profile and outpatient deliverability relative to the existing local radionuclide option, [^131^I]I-mIBG therapy [2,5,13-15], make it a plausible intensification strategy that does not presuppose access to transplantation infrastructure. The LuDO-SA trial is therefore best understood not as a version of a HIC trial but as a first-line intensification strategy purpose-built for a setting where the comparator arm already represents the ceiling of currently available standard care.

The trial has several methodological strengths: a prospective, randomized, multisite design across 4 accredited South African academic centers; standardized dosimetry performed and reconstructed centrally (Tygerberg Hospital, using a single calculation algorithm) to ensure comparability of absorbed-dose estimates across sites; blinded, adjudicated response scoring despite the trial being open-label; and a genuinely novel first-line application of [^177^Lu]Lu-DOTATATE PRRT, addressing a treatment setting with no existing published data. The protocol is also explicit about its feasibility-driven design, providing a transparent sample-size rationale rather than presenting an underpowered comparison as if it were confirmatory.

Several limitations should be acknowledged. The sample size (10 patients per arm) is small, reflecting both the rarity of stage 4 HR-NB in South Africa (approximately 30 new cases nationally per year) and the trial’s feasibility and safety objective; as a result, efficacy estimates will carry wide CIs and cannot support definitive conclusions about comparative benefit. Blinding of participants, families, and treating clinicians is not possible, given the nature of the intervention (a radioisotope infusion with a distinct admission and monitoring pathway), which could introduce ascertainment bias into nonblinded aspects of care, though the primary response assessment itself is blinded and adjudicated. The trial is conducted in a single country, which limits the generalizability of feasibility findings (staffing, isotope logistics, and accommodation support) to other LMIC settings with different infrastructures. Finally, the design does not include a head-to-head comparison with [^131^I]I-mIBG therapy, the existing local radionuclide standard for induction intensification: the standard arm reflects chemotherapy alone, so the LuDO-SA trial can establish whether [^177^Lu]Lu-DOTATATE improves on chemotherapy alone but not how it compares with the current alternative radionuclide approach.

In the event the LuDO-SA trial confirms both an acceptable DLT profile in the experimental arm per the prespecified stopping rule and a numerically favorable trend in end-of-induction mCR relative to the standard arm, the findings would support progression to a larger trial. A larger trial should be an adequately powered randomized controlled trial with metastatic response rate as the formally powered primary end point rather than toxicity and feasibility. Such a trial should ideally be multinational to achieve adequate accrual given the rarity of the disease. Conversely, if the DLT stopping rule is triggered or no meaningful trend in mCR is observed, it would argue against further first-line development of single-dose [^177^Lu]Lu-DOTATATE at this activity and scheduling. A new randomized trial with adjustments to dose, timing, or patient-selection refinements should be evaluated before any further first-line trial is considered. In settings where more robust molecular diagnostics are available, randomizations can be planned between patients with positive and negative molecular diagnostics, for example, *MYCN* and *ALK*-gene mutations at diagnosis.

Trial results will be disseminated to participants, health care professionals, the public, and other relevant stakeholders through reporting in the trial registry; publication in an open-access, peer-reviewed journal; presentation at relevant academic congresses and meetings; and inclusion as part of a doctoral thesis at the University of Antwerp, Belgium. Annual progress reports will be submitted to the SAHPRA. Authorship of resulting publications will follow standard authorship eligibility guidelines. Data will be shared upon reasonable request, within the terms and regulations set by the participating ethics committees.

### Practical Considerations

#### Radiation Monitoring

The experimental arm contains a single administration of [^177^Lu]Lu-DOTATATE PRRT. Each site is Department of Health–accredited to administer radio-nuclear isotopes. The postadministration care for the single administration will be done according to these guidelines (see RN787: guidelines are similar to [^131^I]I-isotopes in Multimedia Appendix 9). Each site has a qualified, board-certified radio-nuclear physicist and nuclear physician to oversee this care.

Independent of the administration of a single dose of [^177^Lu]Lu-DOTATATE PRRT, patients will be treated according to a standard-of-care national protocol for neuroblastoma.

Therefore, participants will receive extensive pretrial and posttrial care.

#### Trial Communications

For participating trial units, protocol-related communications and training will be provided by the national PIs on a semiannual basis or for urgent communications on an ad hoc basis via electronic communications.

Protocol-related deviations or serious adverse events will be communicated by local PIs to their respective ethics committees. Progress reports will be submitted by the national PIs to the relevant regulatory agencies, trial registries, and ethics committees.

## Supplementary material

10.2196/93547 Multimedia Appendix 1 Comparison between nuclear radiotherapy and autologous stem cell transplant.

10.2196/93547 Multimedia Appendix 2 International Neuroblastoma Response Criteria.

10.2196/93547 Multimedia Appendix 3 SPIRIT (Standard Protocol Items: Recommendations for Interventional Trials) figure of the experimental arm.

10.2196/93547 Multimedia Appendix 4 Medical contraindications for induction radioisotope therapy.

10.2196/93547 Multimedia Appendix 5 Data collection sheet.

10.2196/93547 Multimedia Appendix 6 Induction chemotherapy for high-risk neuroblastoma according to the South African Children's Cancer Study Group (SACCSG) NB-2017 protocol.

10.2196/93547 Multimedia Appendix 7 The diagnostic investigations in the standard and experimental (interventional) arm.

10.2196/93547 Multimedia Appendix 8 Camera setup and imaging procedures during ^177^[^177^Lu]Lu-DOTATATE therapy.

10.2196/93547 Multimedia Appendix 9 RN787: guidelines are similar to I-131 isotopes.

10.2196/93547 Checklist 1 SPIRIT checklist.

## Abbreviations

[^131^I]I-mIBG: [^131^I]Iodine metaiodobenzylguanidine
[^177^Lu]Lu-DOTATATE: [^177^Lu]Lutetium DOTATATE
[^68^Ga]Ga-DOTA-peptide: [^68^Ga]Gallium DOTA-peptide
CT: computed tomography
CTCAE: Food and Drug Administration’s Common Terminology Criteria for Adverse Events
DLT: dose-limiting toxicity
DSMC: Data and Safety Monitoring Committee
HIC: high-income country
HR-NB: high-risk neuroblastoma
HVA: homovanillic acid
INRC: International Neuroblastoma Response Criteria
LMIC: low- and middle-income country
mCR: metastatic complete remission
OJEC: Vincristine, Carboplatin, Etoposide, and Cyclophosphamide
OPEC: Vincristine, Cisplatin, Etoposide, and Cyclophosphamide
OS: overall survival
PET: positron emission tomography
PI: principal investigator
POPI: Protection of Personal Information
PRRT: peptide receptor radionuclide therapy
rCOJEC: rapid cisplatin, vincristine, etoposide, cyclophosphamide, and carboplatin
RECIST: Response Evaluation Criteria in Solid Tumors
SACCSG: South African Children's Cancer Study Group
SAHPRA: South African Health Products Regulatory Authority
SPECT: single-photon emission computed tomography
SPIRIT: Standard Protocol Items: Recommendations for Interventional Trials
SSTR: somatostatin receptor
VMA: vanillylmandelic acid
