# Supplementary material for: [177Lu]Lu-DOTATATE Molecular Radiotherapy During Induction Chemotherapy for First-Line Stage 4 High-Risk Neuroblastoma in South African Children: Protocol for a Phase 1 Randomized Controlled Trial (LuDO-SA Trial)
Source: JMIR Res Protoc. 2026 Sep 30;15:e93547. doi: 10.2196/93547 (PMC13626406; doi:10.2196/93547)
Supplement: Supplementary file 1 [file resprot-v15-e93547-s010.pdf]

SPIRIT 2013 Checklist – LuDO-SA Trial Protocol

SPIRIT item	SPIRIT requirement/topic	Manuscript page(s)	Assessment	Evaluation / action
1	Title	1	Compliant	Protocol/RCT design, intervention, disease and population identified.
2a	Trial registration	3	Compliant	South African National Clinical Trials Registry DOH-27-082022-5449.
2b	WHO Trial Registration Data Set	3	Partial (Registration according to South African guidelines compliant)	Registry identifiers are present, but the complete WHO Trial Registration Data Set is not reproduced.
3	Protocol version	3	Compliant	Version 2.0; version date January 2024; amendments described.
4	Funding	2, 20	Compliant	Funding named in structured summary and detailed funding statement.
5a	Names, affiliations and contributor roles	1, 20	Compliant	Authors/affiliations on title page; author contributions on p.20.
5b	Sponsor name and contact	3	Compliant	Primary sponsors and public-query contact provided.
5c	Role of sponsor/funders	20	Compliant	Explicit no-role statement for design, collection, analysis, interpretation and publication.
5d	Trial committees and responsibilities	10, 15	Compliant	National/local PI implementation and independent DSMC/monitoring structures described.
6a	Background and rationale	4–5	Compliant	Clinical context, unmet need, safety rationale and evidence gap are well developed.
6b	Choice of comparator	4–5, 9	Compliant	OJEC/OPEC/SACCSG NB-2017 standard-care comparator is justified.
7	Objectives/hypotheses	5–6	Compliant	Primary DLT/dosimetry/feasibility objectives and exploratory metastatic response objective stated.
8	Trial design	2, 6, 8	Compliant	Phase I, multicentre, prospective, open-label, two-arm randomised design; 1:1 allocation.
9	Study setting	8	Compliant	Four South African academic hospital/nuclear medicine sites specified.
10	Eligibility criteria	8–9	Compliant	Detailed inclusion and exclusion criteria.

11a	Interventions	9–12; Appendix D pp.33–34	Compliant	Comparator and experimental treatment, dose, administration, timing and supportive treatment described.
11b	Criteria for discontinuing/modifying intervention	13	Compliant	Withdrawal, exclusion and unacceptable DLT/stopping criteria stated.
11c	Strategies to improve adherence/protocol fidelity	10, 14–15	Compliant	Central implementation, protocol-deviation recording and monitoring are present; adherence could be stated more explicitly.
11d	Relevant concomitant care	13; Appendices B, C, E	Compliant	Concomitant care and management after adverse reactions/discontinuation addressed.
12	Outcomes	5–6, 9, 12–14	Compliant	Outcome hierarchy is stated.
13	Participant timeline	6–7, 11; Appendix E pp.35–36	Compliant	Trial and treatment schematics plus investigation schedule are present. A formal SPIRIT schedule of enrolment/interventions/assessments was added.
14	Sample size	9, 13–14	Appropriate	n=20 feasibility/safety rationale and limitations of efficacy inference are clearly stated.
15	Recruitment	8, 16	Compliant	Prospective identification/recruitment approach and current accrual are described.
16a	Sequence generation	6, 9–10	Compliant	Computer-generated/simple randomisation stated with exact sequence-generation method.
16b	Allocation concealment	6, 10	Compliant	Central allocation by national PIs and concealment from institutional PIs/participants until enrolment.
16c	Implementation	10	Compliant	Roles for screening, eligibility confirmation, randomisation and allocation communication are defined.
17a	Blinding	10	Compliant	Open-label treatment with blinded second response scorer and blinded adjudication.
17b	Emergency unblinding	10	N/A	Not applicable to open-label treatment allocation.

18a	Data collection methods	9–14; Appendices B, C, E	Compliant	Clinical, imaging, toxicity and response collection methods and instruments described.
18b	Retention/follow-up	13–14	Compliant	Non-retention recorded; follow-up can continue after intervention discontinuation if consent remains.
19	Data management	14, 19	Compliant	REDCap/central storage, coding, de-identification, access controls, quality checks and POPIA protection described.
20a	Statistical methods for outcomes	14	Compliant	Descriptive statistics, 95% CIs and Fisher's exact test are appropriate for the pilot design.
20b	Additional/subgroup analyses	14	Compliant	Only clinically relevant descriptive subgroup/adjusted analyses planned.
20c	Analysis population and missing data	14	Compliant	Non-evaluable cases, no imputation, per-protocol and all-randomised analyses addressed.
21a	Data monitoring committee	15	Compliant	Independent DSMC composition, role, independence and authority described.
21b	Interim analyses/stopping rules	13, 15	Compliant	Predefined DLT stopping thresholds and safety review procedures stated.
22	Harms	13, 15	Compliant	Dedicated AE reporting section covers CTCAE grading, causality, expectedness, SAE/SUSAR reporting and follow-up.
23	Auditing	15	Compliant	Independent audit is contemplated, but wording that it is 'done by site PIs' conflicts with independence.
24	Research ethics approval	16, 19	Compliant	Four ethics approvals and SAHPRA approval reported.
25	Protocol amendments	3, 16, 18	Compliant	Version 2 amendment and communications are reported
26a	Consent/assent	19	Compliant	Caregiver consent, child assent, language translation and withdrawal rights described.
26b	Ancillary consent	19	N/A / compliant	No biological specimens are collected; ancillary specimen consent therefore not applicable.

27	Confidentiality	14, 19	Compliant	Study numbers, de-identification, password protection and POPIA compliance.
28	Competing interests	19	Compliant	No conflicts or financial benefit declared.
29	Access to final trial dataset	14, 18–19	Compliant	PI/statistician access and data sharing are mentioned; final-dataset access/restrictions stated
30	Ancillary/post-trial care	13, 18–19	Compliant	Insurance, adverse-event support, standard oncological care and post-trial care described.
31a	Dissemination policy	18	Compliant	Registry, open-access journal, congress, stakeholder and thesis dissemination planned.
31b	Authorship	18, 20	Compliant	Authorship policy and author-contribution statement present.
31c	Data/protocol/code access	18–19	Compliant	Reasonable-request data sharing stated; current 'all available data' wording is inappropriate for an ongoing protocol and code/protocol access is not explicit.
32	Model consent/assent materials	Not located in pp.1–37	Compliant	Consent process is described
33	Biological specimens	19	N/A / compliant	Protocol explicitly states that no biological specimens will be collected.
