# Supplementary material for: [177Lu]Lu-DOTATATE Molecular Radiotherapy During Induction Chemotherapy for First-Line Stage 4 High-Risk Neuroblastoma in South African Children: Protocol for a Phase 1 Randomized Controlled Trial (LuDO-SA Trial)
Source: JMIR Res Protoc. 2026 Sep 30;15:e93547. doi: 10.2196/93547 (PMC13626406; doi:10.2196/93547)
Supplement: Supplementary file 2 [file resprot-v15-e93547-s001.docx]

**Supplemental table S1:** Comparison between nuclear radiotherapy and autologous stem cell transplant

| **Criteria** | **[^177^Lu]Lu-DOTA-TATE therapy** | **[^131^I]mIBG therapy** | **ASCT** |
| --- | --- | --- | --- |
| Up front therapy | Yes | Yes | No |
| Admission | 1 day | Multiple days | Weeks |
| Special admission | No | Lead room | Isolation transplantation ward |
| Availability | Predictable  Can be imported | Inconsistent | Requires mCR and limited resources |
| Upfront investigations | Standard of care  mGFR | Standard of care  mGFR  Additional pre-toxicity evaluations | Multiple investigations for transplant eligibility  mGFR  Additional pre-toxicity evaluations |
| Preparation | Single arginine/and lysine infusion before therapy | Thyroid protection with iodine preparations | Apheresis  Screening |
| Pre infusion medication | Yes | Yes | Yes |
| Safety and supportive care | Acceptable toxicity | High toxicity in need of supportive care including G-CSF | Myelo-ablative toxicity with high need for supportive care over a period |
| Price of procedure (Based on 2022 prices) | ZAR 16,750.00 – ZAR24,500.00  [US $1,025.00 – US $1,500.00] | ZAR25,000.00  [US $1,550.00] | ZAR 500,000.00 [US$ 30,600.00] |
| Data in children | Limited | Yes | Extensive |
