# Supplementary material for: [177Lu]Lu-DOTATATE Molecular Radiotherapy During Induction Chemotherapy for First-Line Stage 4 High-Risk Neuroblastoma in South African Children: Protocol for a Phase 1 Randomized Controlled Trial (LuDO-SA Trial)
Source: JMIR Res Protoc. 2026 Sep 30;15:e93547. doi: 10.2196/93547 (PMC13626406; doi:10.2196/93547)
Supplement: Supplementary file 3 [file resprot-v15-e93547-s002.docx]

**Appendix A:** International Neuroblastoma Response Criteria

**Abbreviations:**

CR – Complete response; MD – Minimal disease; MR – Minimal response; NI – not involved;

PD – progressive disease; PR – partial response; VGPR – very good partial response;

SD – stable disease

**Definitions:**

**Target lesions:** Disease sites that meet criteria of measurable size (non-lymphoid soft tissue mass ≥10 mm in longest dimension or lymph node ≥15mm in short axis) as well as either uptake on MIBG OR biopsy positive for neuroblastoma or ganglioneuroblastoma

**Non-target lesions:** Lesions that are considered to be active tumour sites but do not meet target lesion criteria including leptomeningeal tumour, tumour in cerebrospinal fluid, ascites, and pleural effusion cytology.

| **Primary (soft tissue) Tumour Response** | |
| --- | --- |
| **Response** | **Anatomic + [^123^I]-MIBG and/ or [^68^Ga]Ga DOTA PET/CT** **Imaging** |
| **CR** | Assessment with:   - 10 mm residual soft tissue at primary site   **AND**   - Complete resolution of [^123^I]-MIBG uptake at primary site.   If no [^123^I]-MIBG and/ or [^68^Ga]Ga DOTA PET/CT scan available:   - 10 mm residual soft tissue at primary site   **AND**   - Catecholamines are normal. |
| **VGPR** | Assessment with:   - Primary tumour decreased by 90% to 99%   **AND**   - catecholamines normal.   Supporting evidence:   - Residual [^123^I]-MIBG and/ or [^68^Ga]Ga DOTA PET/CT scan uptake at primary site. |
| **PR** | Assessment with:   - 30% decrease in longest diameter of primary site   **AND**   - [^123^I]-MIBG and/ or [^68^Ga]Ga DOTA PET/CT scan uptake at primary site stable, improved, or resolved. |
| **PD** | Assessment with:   - 20% increase in longest diameter taking as reference the smallest sum on study (this includes the baseline sum if that is the smallest on study)   **AND**   - Minimum absolute increase of 5 mm in longest dimension |
| **SD** | Neither sufficient shrinkage for PR **nor**  sufficient increase for PD at the  primary site |

| **Tumour Response at Metastatic Soft Tissue and Bone Sites** | |
| --- | --- |
| **Response** | **Anatomic + [^123^I]-MIBG and/ or [^68^Ga]Ga DOTA PET/CT scan** **Imaging** |
| **CR** | Resolution of all sites of disease defined as:   - Non-primary target and non-target lesions measure 10 mm   **AND**   - Lymph nodes identified as target lesions decrease to a short   axis 10 mm  **AND**   - [^123^I]-MIBG uptake and/ or [^68^Ga]Ga DOTA PET/CT scan uptake of non-primary lesions resolves completely   If no [^123^I]-MIBG and/ or [^68^Ga]Ga DOTA PET/CT scan scan available:   - Resolution of all sites   **AND**   - Catecholamines are normal. |
| **VGPR** | Assessment with:   - No tumour   **AND**   - Catecholamines normal   **AND**   - Residual [^123^I]-MIBG and/ or [^68^Ga]Ga DOTA PET/CT scan |
| **PR** | Assessment with:   - >30% decrease in sum of diameters of non-primary target lesions compared with baseline   **AND**   - All of the following: - Non-target lesions may be stable or smaller in size AND - No new lesions AND - >50% reduction in MIBG absolute bone score (relative [^123^I]-MIBG bone score > 0.1 to < 0.5) or > 50% reduction in number of and [^68^Ga]Ga DOTA PET/CT scan –avid bone lesions |
| **PD** | Any of the following:   - Any new soft tissue lesion detected by CT/MRI that is also [^123^I]-MIBG avid and/ or [^68^Ga]Ga DOTA PET/CT scan avid - Any new soft tissue lesion seen on anatomic imaging that is biopsied and confirmed to be Neuroblastoma or Ganglioneuroblastoma - Any new bone site that is [^123^I]-MIBG avid - A new bone site that is [^68^Ga]Ga DOTA PET/CT scan **AND** has CT/MRI findings consistent with tumour **OR** has been confirmed histologically to be Neuroblastoma or Ganglioneuroblastoma - 20% increase in longest diameter taking as reference the smallest sum on study (this includes the baseline sum if that is the smallest on study) **AND** minimum absolute increase of 5 mm in sum of diameters of target soft tissue lesions - Relative MIBG score > 1.2 |
| **SD** | Neither sufficient shrinkage for PR nor sufficient increase for  PD of non-primary lesions |

| **Bone Marrow Metastasis Response** | |
| --- | --- |
| **Response** | **Cytology/Histology** |
| **CR** | Bone marrow with no tumour infiltration on reassessment,  independent of baseline tumour involvement. |
| **PD** | Any of the following:   - Bone marrow without tumour infiltration that becomes > 5% tumour infiltration on reassessment OR - Bone marrow with tumour infiltration that increases by > two fold   and has > 20% tumour infiltration on reassessment. |
| **MD** | Any of the following:   - Bone marrow with < 5% tumour infiltration and remains > 0 to < 5% tumour infiltration on reassessment **OR** - Bone marrow with no tumour infiltration that has < 5% tumour infiltration on reassessment **OR** - Bone marrow with > 20% tumour infiltration. |
| **SD** | Bone marrow with tumour infiltration that remains positive with >5% tumour infiltration on reassessment but does not meet CR, MD, or PD criteria. |

| **Determination of Overall Response** | |
| --- | --- |
| **Response** | **Criterion** |
| **CR** | All components meet criteria for CR. |
| **PR** | PR in at least one component and all other components are either CR, MD (bone marrow), PR (soft tissue or bone), or NI; no component with PD. |
| **MR** | PR or CR in at least one component but at least one other component with SD; no component with PD. |
| **SD** | SD in one component with no better than SD or NI in any other component; no component with PD. |
| **PD** | Any component with PD. |

Reference:

1. Brodeur, G; Pritchard, J; Berthold, F; Carlsen, N; Castel, V; Castelberry, R; et al. Revisions of the international criteria for neuroblastoma diagnosis, staging, and response to treatment. *J Clin Oncology* 1993;11(8):1466– 77
2. Park, J; Bagatell, R; Cohn, S; Pearson, A; Villablanca, J; Berthold, F; Burchill, S; Boubaker, S; McHugh, K; Nuchtern, J; London, J; Seibel, N; Lindwasser, O; Maris, J; Brock, P; Schleiermacher, G; Ladenstein, R; Matthay, K; Valteau-Couanet, D. Revisions to the International Neuroblastoma Response Criteria: A Consensus Statement From the National Cancer Institute Clinical Trials Planning Meeting. J Clin Oncol 2017; 35(22):2580-2587.
3. Eisenhauer; E, Therasse, P Bogaert, J; Schwartz, L; Sargent, D; Ford, R; Dancey, J; Arbuck, S; Gwyther, S; Mooney, M; Rubinstein, L; Shankar, L; Dodd, L; Kaplan, R; Lacombe, D; Verweij, J. New response evaluation criteria in solid tumours: Revised RECIST guideline (version 1.1). European Journal of Cancer 2009; 45(2): 228-247
