# Supplementary material for: [177Lu]Lu-DOTATATE Molecular Radiotherapy During Induction Chemotherapy for First-Line Stage 4 High-Risk Neuroblastoma in South African Children: Protocol for a Phase 1 Randomized Controlled Trial (LuDO-SA Trial)
Source: JMIR Res Protoc. 2026 Sep 30;15:e93547. doi: 10.2196/93547 (PMC13626406; doi:10.2196/93547)
Supplement: Supplementary file 4 [file resprot-v15-e93547-s004.docx]

**Appendix B:** Medical contra-indications for induction radio-isotope therapy

Urgent need for chemotherapy or surgery due to neuroblastoma complications as defined in the SACCSG NB-2017 study protocol:

- Patients with sepsis or untreated blood culture positive or progressive infections as assessed by radiographic studies.
- Respiratory distress due to hepatic enlargement
- Respiratory failure defined by oxygen requirement or carbon dioxide retention exceeding 60mmHg
- Circulatory failure defined by hypotension or hypertension according to the age specific blood pressure reference values.
- Hepatic failure defined by grade 3 toxicity of bilirubin, fibrinogen, or thrombin time.
- Renal failure defined by impaired blood urea or creatinine, new development of hydronephrosis or deteriorating pre-existent hydronephrosis.
- Symptomatic or asymptomatic intraspinal involvement documented by MRI
- Failure of other organ systems.
