# Supplementary material for: [177Lu]Lu-DOTATATE Molecular Radiotherapy During Induction Chemotherapy for First-Line Stage 4 High-Risk Neuroblastoma in South African Children: Protocol for a Phase 1 Randomized Controlled Trial (LuDO-SA Trial)
Source: JMIR Res Protoc. 2026 Sep 30;15:e93547. doi: 10.2196/93547 (PMC13626406; doi:10.2196/93547)
Supplement: Supplementary file 5 [file resprot-v15-e93547-s005.docx]

**Appendix B:** Data collection sheet

| **Information** | | **Classification** | | **Code** |
| --- | --- | --- | --- | --- |
| Source ID code: | |  | |  |
| Sex: | | Female  Male | | F = 0  M = 1 |
| Date of birth (DOB): yy/mm/dd | |  | |  |
| Age (months): | |  | |  |
| Date of diagnosis (DOD):yy/mm/dd | |  | |  |
| Site of primary | | Adrenal  Abdominal (other than adrenal)  Chest  Neck  Primary not found  Other (Specify) | | 0  1  2  3  4  5 |
| Stage (INSS): | | Stage 4S  Stage 1  Stage 2  Stage 3  Stage 4 | | 0  1  2  3  4 |
| Sites of metastasis: | | Bone  Bone marrow  Intra-abdominal  Liver  Lungs  Lymph nodes (Specify)  Paraspinal  Skull  Skin  Other (Specify) | | 0  1  2  3  4  5  6  7  8  9 |
| Therapy | | | | |
| Date of [^177^Lu]Lu-Do therapy administration: yy/mm/dd | |  | |  |
| Dosimetry  SPECT/CT acquisitions:  2 Hours post therapy  24 Hours post therapy  96 Hours post therapy  160 Hours post therapy | |  | |  |
| Tumour response | | | | |
| Primary (soft tissue) Tumour Response | | CR  VGPR  PR  PD  SD | | 0  1  2  3  4 |
| Tumour Response at Metastatic Soft Tissue and Bone Sites | | CR  VGPR  PR  PD  SD | | 0  1  2  3  4 |
| Bone Marrow Metastasis Response | | CR  PD  MD  SD | | 0  1  2  3 |
| Ga68 DOTATATE PET/CT imaging | | Number of sites | | Value |
|  |  | Site description  Uptake less than liver  Uptake equal to liver  Uptake more intense than liver uptake | | 0  1  2 |
|  |  | Bone marrow involvement  Oligometastatic if there are < 5  Lesions or multiple metastases if > than 5 | | 0  1 |
| Toxicity | | | | |
| Haematological | H1 Haemoglobin  H2 Leukocytes  H3 Granulocytes  H4 Platelets  H5 Haemorrhage | | *Grade 0-4 (each)* | |
| Digestive | D1 Bilirubin  D2 AST/ALT  D3 ALP  D4 Amylase  D5 Stomatitis  D6 Nausea/Vomiting  D7 Diarrhoea  D8 Constipation | | *Grade 0-4 (each)* | |
| Metabolic/ renal | M1 Blood creatinine  M2 Proteinuria  M3 Haematuria  M4 Na+  M5 K+  M6 Ca++  M7 Mg++ | | *Grade 0-4 (each)* | |
| Pulmonary | P1 PA02  P2 DL CO  P3 CV  P4 Function | | *Grade 0-4 (each)* | |
| Cardiac | C1 Rhythm  C2 Function  C3 Ischaemia  C4 Echocardiography (FS)  C5 Hypotension  C6 Hypertension | | *Grade 0-4 (each)* | |
| Neurological | N1 Seizures  N2 Cortical  N3 Cerebellar  N4 Sensory  N5 Motor  N6 Vision | | *Grade 0-4 (each)* | |
| Pain | *Specify site* | | *Grade 0-4* | |
| Other | Allergy  Skin  Infection | | *Grade 0-4* | |
| SIOP Boston ototoxicity scales | Audiometry  SNHL = sensorineural hearing loss | | *Grade 0-4* | |
