# Supplementary material for: [177Lu]Lu-DOTATATE Molecular Radiotherapy During Induction Chemotherapy for First-Line Stage 4 High-Risk Neuroblastoma in South African Children: Protocol for a Phase 1 Randomized Controlled Trial (LuDO-SA Trial)
Source: JMIR Res Protoc. 2026 Sep 30;15:e93547. doi: 10.2196/93547 (PMC13626406; doi:10.2196/93547)
Supplement: Supplementary file 6 [file resprot-v15-e93547-s006.docx]

**Appendix C:** Induction chemotherapy for HR-NB according to the SACCSG NB-2017 protocol

**Regimen:**

- Chemotherapy is administered every 21 days for 7 cycles
- Chemotherapy is administered with bone marrow recovery on rising counts of

ANC >1,000 µl and platelets >100,000 µl.

**Doses:**

Vincristine 1.5mg/m^2^ IV stat (max 2mg per dose) x 1 day

Carboplatin 750mg/m^2^ IV over 2 hours x 1 day

Etoposide 175mg/m^2^ IV over 4 hours x 1 day

Cisplatin 80mg/m^2^ IV over 24 hours x 1 day

Cyclophosphamide 1200mg/m^2^ over 1-hour x1 day

**Dose modifications:**

**Infants and low weight children:**

- Body weight below 12kg should be dosed according to their weight (kg) instead of their body surface area (m²) according to the known formula of 30 kg= 1 m^2^.
- Infants with a weight equal and < 5kg:
- The dose in mg/kg **with** a further 1/3 dose reduction is advised.

Vincristine 0.05mg/kg IV stat (max 2mg per dose) x 1 day

Carboplatin 25mg/kg IV over 2 hours x 1 day

Etoposide 5.8mg/kg IV over 4 hours x 1 day

Cisplatin 2.6mg/kg IV over 24 hours x 1 day

Cyclophosphamide 35mg/kg over 1 hour x 1 day

**Precautions:**

*Cisplatin administration:*

- Prehydration for cisplatin

Infused at 200 ml/m²/hr for at least 3 hours with 0.9% sodium chloride with 20 mmol/l potassium chloride

- Posthydration for cisplatin

Infuse at 3 litres/m^2^. To each litre add 10ml KCl (max 20ml), 5ml Ca gluconate (10% solution) or 250mg/L and 2ml (1-2g/L) MgSO_4_. Post hydrate for 12 hours following each cisplatin dose.

- A target urine volume of 400ml/m^2^/6h must be maintained

*Infection support:*

- Timely antimicrobial interventions according to standard guidelines

*Nutritional support:*

- Naso-gastric nutritional support must be started before starting chemotherapy and weekly measurements and monitoring must be done
