# Supplementary material for: [177Lu]Lu-DOTATATE Molecular Radiotherapy During Induction Chemotherapy for First-Line Stage 4 High-Risk Neuroblastoma in South African Children: Protocol for a Phase 1 Randomized Controlled Trial (LuDO-SA Trial)
Source: JMIR Res Protoc. 2026 Sep 30;15:e93547. doi: 10.2196/93547 (PMC13626406; doi:10.2196/93547)
Supplement: Supplementary file 7 [file resprot-v15-e93547-s007.docx]

**APPENDIX E:** The diagnostic investigations in the standard and experimental (interventional) arm

| **Diagnostic tests** | **Timing** | **Standard SACCSG NB-2017 arm** | **[^177^Lu]Lu-DOTATATE therapy arm** |
| --- | --- | --- | --- |
| **Standard of care blood, urine and cerebrospinal fluid tests ٭** | | | |
| Blood tests | At diagnosis, then weekly | X | X |
| Tumour markers | Diagnosis | X | X |
| Urine catecholamines | Diagnosis, post cycle 4 and 7 | X | X |
| Lumbar puncture (only with neurological signs) | Diagnosis | X | X |
| **Standard of care radiological investigations٭** | | | |
| Abdominal ultrasound (in the absence of CT or MRI facilities) | Diagnosis, post cycle 4 and 7 | X | X |
| Chest X-ray AP and lateral (AP only if a CT chest was done) | Diagnosis, post cycle 4 and 7 | X | X |
| CT chest | Diagnosis, post cycle 4 and 7 | X | X |
| CT abdomen or MRI abdomen | Diagnosis, post cycle 4 and 7 | X | X |
| Echocardiography | Diagnosis | X | X |
| Clinical indications for brain and bone metastasis requires pathology directed imaging i.e. CT brain or X-ray of the bones | Diagnosis, post cycle 4 and 7 | X | X |
| A skeletal X-ray screening of the long bones is indicated in units where nuclear skeletal surveillance investigations are not available at the diagnostic phase | Diagnosis, post cycle 4 and 7 | X | X |
| **Standard of care nuclear imaging studies٭** | | | |
| [^123^I]-MIBG/CT scan | Diagnosis, post cycle 4 and 7 | X | X |
| FDG-PET/CT-scan | Diagnosis, post cycle 4 and 7 | X | X  (mIBG non-avid tumours are excluded from the study) |
| [^99m^Tc]Tc- bone scan | Diagnosis | According to institutional protocols | According to institutional protocols |
| Nuclear glomerular filtration rate (nGFR) | Diagnosis, post cycle 4 and 7 | X | X |
| **^177^[177Lu]Lu-DOTATATE therapy arm study investigations** | | | |
| [^68^Ga]Ga-DOTATATE PET/CT scan | Diagnosis, post cycle 4 and 7 | No | X |
| [^99m^Tc]Tc-MAG3-renogram | Diagnosis | No | X |
| **Study dosimetry and treatment response evaluations** | | | |
| [^68^Ga]Ga-DOTATATE PET/CT scan at 24H, 6-8 weeks and end of induction | Peri-[^177^Lu]Lu-DOTATATE molecular radiotherapy | No | X |

٭ Standard of care guidelines based on the National prospective standard of care SACCSG NB-2017 protocol

**Abbreviations:** CT – computed tomography
