# Supplementary material for: [177Lu]Lu-DOTATATE Molecular Radiotherapy During Induction Chemotherapy for First-Line Stage 4 High-Risk Neuroblastoma in South African Children: Protocol for a Phase 1 Randomized Controlled Trial (LuDO-SA Trial)
Source: JMIR Res Protoc. 2026 Sep 30;15:e93547. doi: 10.2196/93547 (PMC13626406; doi:10.2196/93547)
Supplement: Supplementary file 8 [file resprot-v15-e93547-s008.docx]

**MULTIMEDIA APPENDIX 8:** Camera Setup and Imaging procedures during ^177^[177Lu]Lu-DOTA-TATE therapy

1. **Energy Spectra of Lu-177**

The following table define the energy windows to be used during imaging.

| **Energy** | **Photopeak** | **Width** | **Low** | **High** | **PHA low** | **PHA high** |
| --- | --- | --- | --- | --- | --- | --- |
| **208 main** | 208 | 20% | 10% | 10% | 187.2 | 228.8 |

Table 1: Lu-177 energy window.

2. **Lu-177 SPECT/CT Imaging parameters**

Use the following imaging protocol to image patients and for phantom calibration measurements:

| **Parameter** | **Value Recommended** |
| --- | --- |
| **CT** |  |
| Tube Voltage and Tube Current | Low Dose CT |
| Scan Length | Same ac SPECT Scan |
| Matrix | 512 x 512 |
| Zoom | 1 |
| **SPECT** |  |
| Collimator | MEGP |
| Matrix | 128 x 128 |
| Zoom | 1 |
| Scan mode | Continues if available |
| Number of detectors | 2 |
| Detector distance | Auto-contouring |
| Number of projections per detector | 60 (120 in total) |
| Time per projection | 5 seconds |

Table 2: Imaging parameters

3. **Clock Synchronization.**

The clocks of the dose calibrator and the SPECT/CT camera must be synchronized for accurate quantitative measurements. If synchronization is not possible, use a reference clock.

4. **Dose calibrator**

The aim is to be able to accurately measure Lu-177 on the dose calibrator.

- Note the dose calibrator settings for Lu-177:

| Calibration factor: |  |
| --- | --- |

- Note the activity value of the patient vial as stated in the manufacturer certificate:

| Activity (MBq) | Calibration Date | Calibration Time | Serial Number |
| --- | --- | --- | --- |
|  |  |  |  |

- Measure the patient vial and note the activity:

| Activity (MBq) | Measurement Date | Measurement Time |
| --- | --- | --- |
|  |  |  |

- Please ensure that measured activity correspond to activity on the certificate within +-5% when decay correction is taken into account.

5. **Phantom Calibration Measurement**
   5.1. Equipment
        - Phantom: NEMA body phantom with six spheres
        - ~50 MBq Lu-177 for spheres
        - ~300 – 370 MBq Lu-177 for background
        - 200 ml saline bag
        - ~10 Litre water
   5.2. Method
        - Measure the activity in the vial.

|  | Activity (MBq) | Date | Time |
| --- | --- | --- | --- |
| Vial before dispensing |  |  |  |

- Draw ~ 50MBq of Lu-177 from the vial into a syringe and measure in the dose calibrator.

|  | Activity (MBq) | Date | Time |
| --- | --- | --- | --- |
| Syringe before |  |  |  |

- Remove 10 ml of saline from the saline bag.
- Fill the syringe content (~50 MBq) to constitute 10ml. Add the syringe contents to the saline bag and flush the syringe. Measure the residual in the syringe.

|  | Activity (MBq) | Date | Time |
| --- | --- | --- | --- |
| Syringe after |  |  |  |

- Mix the solution in the bag.
- Use the prepared Lu-177 solution to fill the spheres. Close all the spheres.
- Draw ~ 300MBq of Lu-177 from the vial into a syringe and measure in the dose calibrator.

|  | Activity (MBq) | Date | Time |
| --- | --- | --- | --- |
| Syringe before |  |  |  |

- Fill the empty phantom with ~10 l water. Add the syringe contents (~300 – 370 MBq) to the phantom. Measure the residual in the syringe. Close the phantom. Mix well to ensure uniformity.

|  | Activity (MBq) | Date | Time |
| --- | --- | --- | --- |
| Syringe after |  |  |  |

- Acquire SPECT/CT using the parameters of section 1.2
- Add the remaining Lu-177 solution to the empty phantom and fill with pure water.
- Place the phantom on the scanning bed.

| Study start date: |  |
| --- | --- |
| Study start time: |  |

6. **Total activity administered to patient:**

- Measure the activity of the ‘original’ vial received:

|  | Activity (MBq) | Date | Time |
| --- | --- | --- | --- |
| Vial before dispensing |  |  |  |

- Draw up the necessary activity in the syringe.
- Measure the syringe in the dose calibrator.

|  | Activity (MBq) | Date | Time |
| --- | --- | --- | --- |
| Syringe after dispensing |  |  |  |

- Measure the activity in the empty original vial.

|  | Activity (MBq) | Date | Time |
| --- | --- | --- | --- |
| Empty vial after dispensing |  |  |  |

- After infusion into patient, measure the residual activity in the empty syringe:

|  | Activity (MBq) | Date | Time |
| --- | --- | --- | --- |
| Residual in empty syringe |  |  |  |

- Measure the attached lines and three-way tap to determine the residual:

|  | Activity (MBq) | Date | Time |
| --- | --- | --- | --- |
| Residual in empty syringe |  |  |  |

7. **Preparing of reference vial**

- Prepare a Lu-177 reference vial that should be paced in the scanning field of view and imaged with the patient according to the following parameters:
  - 10- 37 MBq
  - 10 ml
- Measure the activity in the reference vial.

|  | Volume (ml) | Activity | Date | Time |
| --- | --- | --- | --- | --- |
| Reference vial |  |  |  |  |

8. **Patient Imaging**

- Patient to be imaged using the parameters in table 1 and 2.
- Patient to be imaged at the following time points: 1-3 h post injection, 20-24, 68-76 and 144-196 hours (scan not interrupted by the weekend or public holiday), or 1-3 h post injection, 20-24, 90-100 and 144-196 hours (scan interrupted by the weekend or public holiday.
- Scan area to be determined by organs of interest for dosimetry.
- Ref vial to be prepared and placed in the scanning field of view.
- Use the worksheet below to as a summary for dosimetry calculations.

| Date: |  |
| --- | --- |
| Prescribed activity: |  |

| ACTIVITY MEASUREMENTS |  |  |
| --- | --- | --- |
| Lu177 Batch No: |  |  |
|  | Activity (MBq) | Time of Measurement |
| Activity administered |  |  |
| Activity in reference vial |  |  |
| Start time of injection |  |  |

| Time point 1: |  |  |
| --- | --- | --- |
| Date and start time of acquisition |  | |
| Time point 2: |  |  |
| Date and start time of acquisition |  | |
| Time point 3: |  |  |
| Date and start time of acquisition |  | |
| Time point 4: |  |  |
| Date and start time of acquisition |  | |

9. **Reconstruction and analysis**

- Reconstruction should be performed with a 3D OSEM algorithm using collimator model if applicable.
- The effective number of iterations (# of iterations multiplied with the number of subsets) should be at least 48 (e.g: 8 subsets, 6 iterations), a 3D Gaussian post filter of 12 mm FHHM or an equivalent frequency cut-off should be applied.
- CT based attenuation correction and TEW scatter correction should be applied.
- Anonymised, reconstructed, as well as raw data and workers’ should be transferred to the Central Dosimetry centre for dosimetry calculations. This includes phantom calibration measurements to determine the sensitivity factor.
- Sensitivity factor will be determined using DOSIsoft software.
